# A Housekeeper's Criticisms on Food by Contract

**Published:** 1912-10-26

**Authors:** 


### INSTITUTIONAL HOUSEKEEPING.

### A Housekeeper's Criticisms on Food by Contract.

In most large hospitals and in all those under State or municipal control contracts for supplies for six or twelve months are the order of the day. Tenders are called for and samples are sent for inspection, and very commonly it is the lowest tender which is accepted. Naturally the samples are the best the would-be contractor can supply at the rate he offers. So far, so good. But here comes the housekeeper's difficulty. She has to provide a specified result with stores which have been chosen with no reference to her. The steward or storekeeper goes over the samples and gives his opinion, which generally decides the matter where it is not the lowest that is chosen merely because it is the lowest. Generally speaking, he knows very little about the quality of the goods to be supplied. Baking-powder is baking-powder to him; that some kinds are reliable and some not does not enter into his reckoning. How should he know? All he has to do is to receive the goods in bulk and hand out the quantity requisitioned for, and once out of his hands (unless he courts indigestion by eating the heavy scones and cakes made with it, and then he blames the cook) he has nothing more to do with the matter. Suppose it is bad; all the answer the worried housekeeper gets when she complains is that baking-powder was contracted for and baking-powder she has got. She must make it do until the case is finished. The result is that, if the housekeeper is very keen on her work, she supplies the reliable sort out of her own pocket instead of requisitioning from the contracted supply which cannot be depended upon. In one instance known to the writer the housekeeper requisitioned large quantities weekly till the amount was finished and threw it away. If she is not very keen she does as well as she can without it, and lets other things go to the wall, as time and material are too valuable to waste on certain failure.

Baking-powder, it need scarcely be said, is a figurative expression meant to stand for a variety of supplies, but it illustrates the point. In some institutions the list of things for which tenders are to be called is made out without any particular kind being specified. The list begins with A—arrowroot, allspice, etc.; bacon, butter, bread, and so on through the alphabet. This is a very slipshod way of doing things. It is most unfair to the steward, who is responsible for the raw material, and to the housekeeper, who is responsible for the finished product. They have to take what is sent with no redress until the contract runs out. The specimen forms of contract supplied in the Uniform System of Accounts has done much to remedy this, but in many parts of the country they are still unknown. The steward is a busy man; the handing out of stores is by no means his chief duty, and he does not want to have the added burden of seeing that things are up to the original sample and returning goods that are not satisfactory. This, if there is any public form of control, means endless correspondence with query clerks as to the "whys and wherefores." Hence he is rather apt to take it for granted that every complaint of the housekeeper is unreasonable.

There is no doubt that the contract list, as far as the food supply is concerned, at any rate, should be submitted to the housekeeper. If she is equal to her post she should be able to inform those responsible what are the best kind of foodstuffs to be obtained for her particular needs. The experienced housekeeper knows that in some cases the most expensive article to start with is the cheapest in the end, but this is by no means always the case.

When special brands have been mentioned in the contract it should be understood that the specified kind only will be received and no others "equally good" from the contractor's point of view will be considered. This saves trouble in the long run to everyone. The contractor will not run the risk of having his goods returned when close supervision is exercised. And the housekeeper is only within her rights when she insists on having the goods contracted for and for which the hospital is paying.

When the contractor finds that the authorities do not seem to know the difference between good and bad but take anything that is sent, the goods very soon fall a long way below sample.

### Housekeeping Queries.

Laundry Matron.—Can you kindly tell me how to remove mildew marks from cotton articles which have been long stored?—This treatment should prove effectual: Wet the stains, rub them thickly over with yellow soap, and cover with a layer of French chalk. Leave out in the sun until the parts have dried, then wet, soap, and chalk again; if necessary repeat a third or fourth time.

